# Donor-derived cell-free DNA testing in pediatric kidney transplant recipients: indications and clinical utility

**DOI:** 10.1007/s00467-025-06770-w

**Published:** 2025-04-14

**Authors:** Jayanthi Chandar, Vaka Sigurjonsdottir, Marissa Defreitas, Tara Gavcovich, Mingming Zhou, Renata Glehn-Ponsirenas, George Burke

**Affiliations:** 1 Department, of Pediatrics, Division of Pediatric Nephrology, University of Miami Miller School of Medicine, Miami, FL USA; 2 Miami Transplant Institute, Jackson Health System, Miami, FL USA; 3 Biostatistics and Data Sciences Department, CareDx, Inc, Brisbane, CA USA; 4 Medical Affairs Department, CareDx, Inc, Brisbane, CA USA; 5 Department of Surgery, Division of Transplantation, University of Miami Miller School of Medicine, Miami, FL USA

**Keywords:** Pediatric kidney transplant recipients, Dd-cfDNA, Biomarker, De novo HLA antibodies, Biopsy proven acute rejection, Immune quiescence

## Abstract

**Background:**

We describe our single-center experience in performing donor-derived cell-free DNA (dd-cfDNA) testing for a clinical indication in pediatric kidney transplant recipients.

**Methods:**

Dd-cfDNA was done for increase in creatinine, appearance of de novo anti-HLA antibodies (*dn*HLAab) and for a clinical indication. We compared clinical characteristics of patients with dd-cfDNA > 1 with those with dd-cfDNA ≤ 1 and also compared dd-cfDNA in patients with no biopsy proven rejection (BPAR) or *dn*HLAab with those with BPAR, and those with *dn*HLAab and no BPAR.

**Results:**

Chart review was performed in 106 patients with a mean age of 11.0 ± 5.5 years. When compared with 62 patients with dd-cfDNA ≤ 1, 59.0% (26/44) of patients with dd-cfDNA > 1 had BPAR (OR 13.5: 95%CI 4.6,38; *p* < 0.0001), and 88.1% (37/44) had *dn*HLAab (OR 60.3 95%CI 17.2,192.2; *p* < 0.0001). Patients with DQ and DR *dn*HLAab (OR 115.2: 95%CI 24.8, 509.5; *p* < 0.0001) and those with donor-specific antibodies (DSAs) (OR 50.8: 95%CI 13.0, 168.7; *p* < 0.0001) were likely to have dd-cfDNA > 1. A repeated measures linear mixed effect model revealed a significant difference in dd-cfDNA between those with no antibodies or BPAR (*p* < 0.0001) and patients with BPAR and *dn*HLAab, with or without DSA. At the end of the follow-up period, eGFR was 72 mL/min/1.73 m^2^ in those without BPAR or *dn*HLAab and was significantly different from those with BPAR (eGFR 51 mL/min/1.73 m^2^ (*p* < 0.0001).

**Conclusions:**

Elevated dd-cfDNA is strongly associated with BPAR, class II *dn*HLAab and DSAs. Conversely, low values are observed in immunoquiescent states. Dd-cfDNA can be a useful tool for non-invasive clinical decision-making.

**Graphical abstract:**

**Figure:**
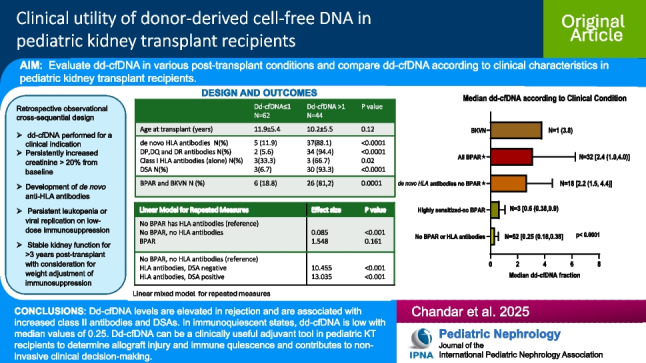
A higher resolution version of the Graphical abstract is available as Supplementary information

**Supplementary Information:**

The online version contains supplementary material available at 10.1007/s00467-025-06770-w.

## Introduction

Long-term kidney transplant survival among pediatric recipients is limited due to complex injurious factors which are both immunologic and non-immunologic [1, 2]. Judicious monitoring for allograft injury and optimization of immunosuppressive therapies are essential for successful long-term outcomes. Preemptive recognition and management of factors that result in kidney injury will be ideal to preserve the life of the kidney transplant. Both rejection and infection lead to acute kidney injury (AKI) [3]. Conventionally, the most used biomarker to determine kidney injury is serum creatinine. Although serum creatinine is used to monitor kidney injury, it may be normal until injury is advanced. In addition, serum creatinine varies with hydration status and calcineurin inhibition levels and has individually established baselines. Moreover, creatinine increases with growth and muscle mass and cannot reliably predict if the increase in creatinine is from growth or kidney injury. Hence, in recent years, several biomarkers have been described for early recognition of AKI in situations such as after cardiac surgery and in high-risk conditions [4]. Sequential monitoring of biomarkers permits early recognition of injury which may allow preventive and therapeutic interventions before irreversible damage occurs [4].

After kidney transplantation, protocols for immunosuppression have traditionally utilized fixed-dose regimens and drug levels to determine immunosuppressive drug doses [5–7]. However, these dosing regimens are not tailored to an individual’s underlying immune status. Furthermore, immunosuppression in pediatric kidney transplant recipients is generally managed by center-specific induction and maintenance protocols which are often derived from those developed for adults [8]. Multiple studies in adult and pediatric patients indicate that donor-derived cell-free DNA (dd-cfDNA) could be an early marker of injury and rejection in the transplanted organ, and a value > 1.0 has been reported to be highly associated with rejection [9–14]. Moreover, in adult cohorts, it has a high negative predictive value when below 0.2% [15]. Dd-cfDNA is performed at regular intervals in the first year after kidney transplantation, and it is considered to be a “liquid biopsy” that can be done in lieu of a protocol biopsy [15]. However, the utility of dd-cfDNA testing in pediatric kidney transplant recipients for conditions other than rejection has not been well-studied. At our institution, we developed a clinical protocol for performing dd-cfDNA testing for a clinical indication in pediatric kidney transplant recipients. We report our single-center retrospective observational study with performing dd-cfDNA for each clinical indication, including association with biopsy-proven rejection (BPAR) and/or de novo anti-HLA antibody. We compared clinical characteristics of patients with dd-cfDNA ≤ 1 versus > 1 and also compared dd-cfDNA in patients with no BPAR or de novo HLA antibodies to those with biopsy proven acute rejection (BPAR) and to patients with de novo HLA antibodies without BPAR.

## Materials and methods

This was a retrospective observational cross-sequential design study. The study was approved by the Institutional Review Board of the University of Miami Human Subjects Research Office and Jackson Health System, IRB # 20,230,722 with a waiver of consent authorization. All subjects were assured anonymity in compliance with the Health Insurance Portability and Accountability Act (HIPAA).

**Fig. 1:**
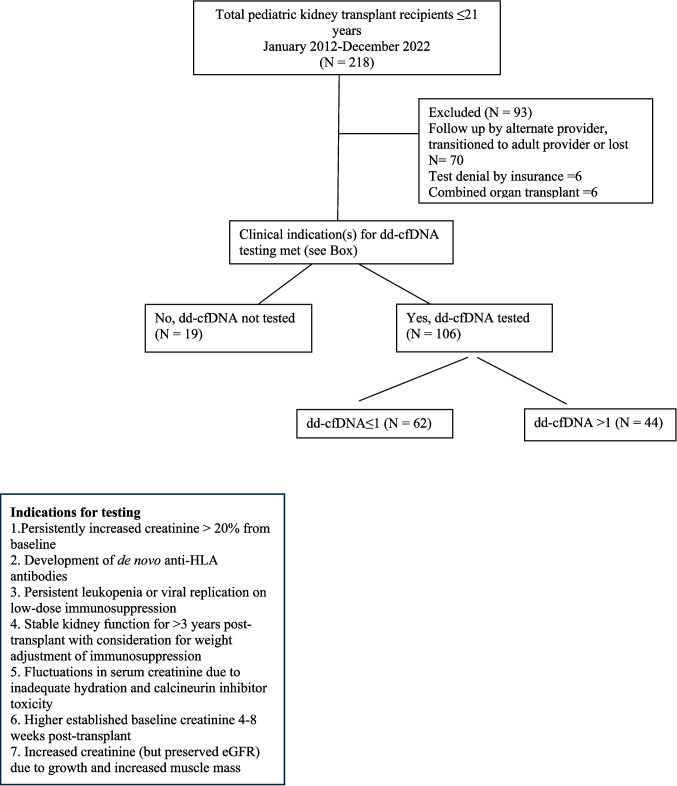
Consort diagram outlining patient selection criteria for dd-cfDNA testing

As seen in the consort diagram in Fig. 1, from a total of 218 patients transplanted from January 2012 to December 2022, cross-sectional data was obtained in 106, and longitudinal data with at least two values of dd-cfDNA were available in 97 children seen at the post-transplant clinic at the Miami Transplant Institute from January 2020 to March 2023. Data collected included patient demographics, dd-cfDNA with longitudinal data when available, serum creatinine at the time of collection of dd-cfDNA, cystatin C and tacrolimus levels, results of kidney biopsy done for a clinical indication, calculated panel reactive antibody (cPRA), anti-HLA donor-specific antibodies (DSA), results of cytomegalovirus (CMV), Epstein Barr virus (EBV), and BK virus (BKV) polymerase chain reaction (PCR), and height at each time-point of sample collection.

*Donor-derived cell-free DNA testing*: Donor-derived cell-free DNA (dd-cfDNA) was measured by a targeted next-generation sequencing assay that utilizes single-nucleotide polymorphisms for the quantification of dd-cfDNA, without the necessity for distinct genotyping of the recipient or donor. Five milliliters of venous blood was collected in Streck Cell-Free DNA BCT tubes and transported to CareDx, Inc.’s central Clinical Laboratories Improvements Act-certified laboratory. The methodology for standardized specimen processing and analytical techniques, aimed at determining the percentage of dd-cfDNA, has been previously described elsewhere [16, 17].

As seen in the box in Fig. 1, all patients with persistently increased creatinine > 20% from baseline and those who developed de novo anti-HLA antibodies had dd-cfDNA testing done. In addition, dd-cfDNA testing was done for the following clinical indications:

1. Patients with leukopenia or persistent viral replication who were on low-dose immunosuppression
2. Those with stable kidney function for > 3 years and for whom weight adjustment for immunosuppression was being considered
3. Patients with fluctuations in serum creatinine due to inadequate hydration and calcineurin inhibitor toxicity
4. Those with higher established baseline creatine
5. Those with increased creatinine (but preserved eGFR) due to growth and increased muscle mass. Some patients had more than one clinical indication for testing.

BPAR was defined as outlined and classified by the BANFF 2019 criteria [18]. The biopsies were categorized as normal, borderline, acute 1A T-cell-mediated rejection (TCMR), acute 1B TCMR, and active antibody-mediated rejection (ABMR). Biopsies done within a 3-month timeframe of dd-cfDNA testing were included in this study.

CPRA and DSA were performed once a month for the first 6 months; if negative, once every 2 months from 7–12 months; if negative, once every 3 months for 3 years and once every 6 months after the third post-transplant year.

Our induction protocol since 2020 is 1 mg/kg of thymoglobulin (3 mg/kg prior to 2020), 2 doses of basiliximab and methylprednisolone taper. A steroid-free protocol is achieved in patients when tacrolimus levels are therapeutic, in those who are not sensitized, have autoimmune disease, or have an uncomplicated course. Our target trough levels for tacrolimus in the first 6 months after transplant are 6–8 ng/mL and 5–7 ng/mL until the third post-transplant year. Thereafter, the trough level is maintained between 4 and 6 ng/mL.

The dose of mycophenolate is 600 mg/m^2^/dose. Dose adjustments are made based on white blood cell count and gastrointestinal side-effects. A maintenance dose of < 450 mg/m^2^/dose is considered low maintenance dosing.

Low immune suppression was defined as the need for lowering immunosuppression from baseline at > 1 month after transplant because of leukopenia ≤ 3500 white cells/µL of blood, or replication of CMV, EBV, parvovirus, adenovirus, or BKV by PCR ≥ 137 IU/mL. Patients who continued to receive lower maintenance immune suppression than described in published studies (> 3 years after transplant) were also included in this category [8].

The glomerular filtration rate was estimated (eGFR) with the under 25 (U25) equation as validated by the Chronic Kidney Disease in Children (CKiD) study [19]. The equation combines serum creatinine and cystatin C to estimate GFR and is given by the equation: GFR = K × [height (m)/serum creatinine (mg/dL)] + [K Cystatin C/Serum Cystatin C (mg/L)]/2. The constant *K* varies with sex and age [19].

The primary outcomes of the study were (1) to compare clinical characteristics of pediatric kidney transplant recipients with dd-cfDNA > 1 with those with dd-cfDNA < 1 and (2) to compare dd-cfDNA in patients with no BPAR or de novo HLA antibodies versus those with BPAR versus those with de novo HLA antibodies with no BPAR. A secondary outcome was to compare longitudinal measurements of eGFR in the latter 3 categories of patients.

*Statistical analysis*: Categorical variables were analyzed by chi-square analysis or Fisher *T* test. Dd-cfDNA was not normally distributed, and hence, the median values were used for analysis. Dd-cfDNA values for each clinical indication and type of BPAR were compared with the Kruskal Wallis test. Post hoc testing was performed with Dunn’s multiple comparisons test. The median value of patients with dd-cfDNA ≤ 1 was compared with those with dd-cfDNA > 1 using the Mann–Whitney test. Sequential values of Dd-cfDNA and eGFR were compared between patients who had never had BPAR or de novo anti-HLA antibodies versus those with no clinical signs of rejection but had de novo HLA antibodies (defined as those with no decline in eGFR but had presence of de novo HLA antibodies) versus those with BPAR including borderline rejection with a linear mixed effect model for repeated measures. Statistical analysis and graphs were performed using GraphPad® Prism version 10.3., and linear mixed models were generated in R 4.3.0.

## Results

### Clinical and demographic characteristics

The age, gender, race, and mean/median time to testing of the 106 children in this cohort are displayed in Table 1. There was a male predominance, and the cohort was of diverse race and ethnic background with Hispanic ethnicity (50%) and African American race (33%) being most prevalent. There were a higher proportion of White Hispanics with dd-cfDNA ≤ 1 (*p* = 0.03).

**Table 1:** Demographics and patient characteristics

	All	Dd-cfDNA ≤ 1.0	Dd-cfDNA > 1.0	*p* value
Number of children	106	62	44	-
Age at transplant (years)	11 ± 5.5	11.9 ± 5.4	10.2 ± 5.5	0.12
Sex	70 M 36 F	M 41 F 21	29 M 15 F	1.0
Race^1^				0.03
White non-Hispanic ***N*** (%)	14 (13.2)	9 (64.3)	5 (35.7)	
White Hispanic ***N*** (%)	50 (47.2)	35 (70.0)	15 (30.0)	
Black Hispanic ***N*** (%)	3 (2.8)	1(33.0)	2 (67.0)	
African American ***N*** (%)	36 (34.0)	15 (41.7)	21 (58.3)	
Asian ***N*** (%)	3 (2.8)	2 (67.0)	1 (33.0)	
Mean time to test from transplant (years)	3.5 ± 3.7	3.3 ± 3.9	3.9 ± 3.4	0.42
Median (IQR)	2.1 (0.79,5)	1.5 (0.6,4.5)	3.5 (1.0,5.6)	
De novo HLA antibodies ***N*** (%)	42 (39.6)	5 (11.9)	37 (88.1)	< 0.0001
DP, DQ, and DR ***N*** (%)	36 (34.0)	2 (5.6)	34 (94.4)	< 0.0001
Class I alloantibodies (alone) ***N*** (%)	6 (5.7)	3 (33.3)	3 (66.7)	0.02
Positive DSA ***N*** (%)	33 (28.3)	3 (6.7)	30 (93.3)	< 0.0001
Highly sensitized ***N*** (%)	5 (4.7)	3 (60)	2 (40)	0.7
TCMR 1 A, 1B, ABMR, borderline rejection and BKVN ***N*** (%)	32 (30.2)^2^	6 (18.8)^3^	26 (81.2)^4^	< 0.0001

^1^Black Hispanic and Asian were not included in the analysis due to small numbers

^2^Of the 32, 5 had 2 biopsies

^3^Seven biopsies were performed in 6 patients, 3 revealed borderline rejection, 2 TCMR 1 A, and 2 TCMR 1B

^4^Thirty biopsies were performed in 26 patients; 12 had TCMR 1 A, 6—TCMR 1B, 5—ABMR, 6—borderline rejection, and 1—BK nephropathy

Parenthesis in “all” categories represent column percentages; parenthesis for Dd cfDNA ≤ 1 and > 1 represent row percentages

**Fig. 2:**
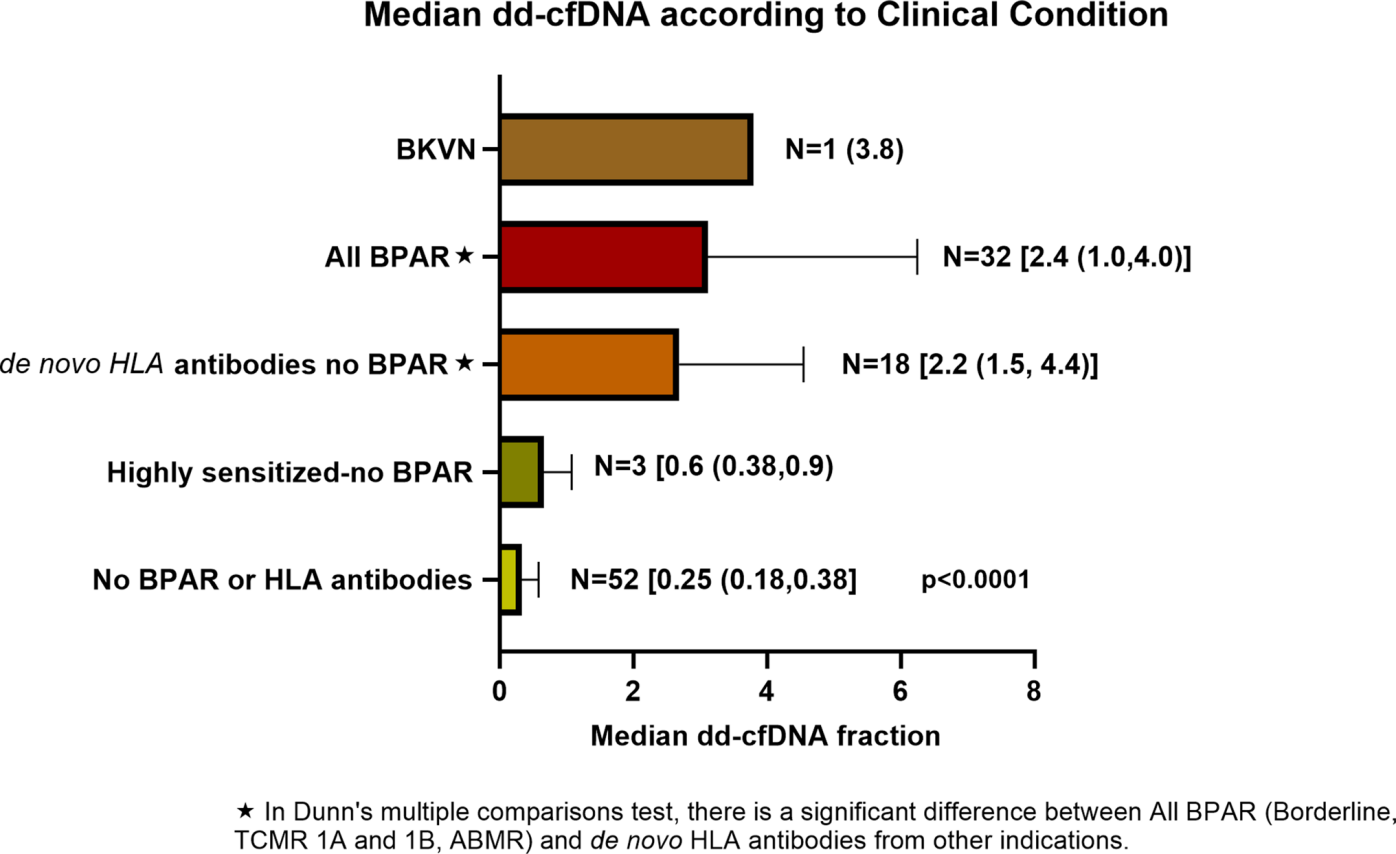
Median dd-cfDNA with IQR in parathesis in different clinical scenarios. Patients with BPAR which included TCMR 1B, 1 A, and ABMR and borderline rejection and those with de novo HLA antibodies had higher values of dd-cfDNA when compared to 52 patients with no BPAR or who had not developed anti-HLA antibodies (the values of dd-cfDNA measured at the time of BPAR or development of de novo anti-HLA antibodies were used in the patients who had BPAR or de novo anti-HLA antibodies)

Figure 2 depicts dd-cfDNA fractions in all patients based on the presence or absence of BPAR and history of sensitization or presence of anti-HLA antibodies without clinical signs of rejection. There were 136 dd-cfDNA values in 52 patients with no BPAR or de novo HLA antibodies. Their dd-cfDNA values were consistently low with a median of 0.25 (0.18,0.38; *p* < 0.0001) which in multiple comparisons was significantly different from dd-cfDNA values in patients who had BPAR (ABMR, TCMR 1B, TCMR 1 A, and borderline rejection) and de novo HLA antibodies without BPAR.

### Comparison of patients with dd-cfDNA > 1 versus ≤ 1

Table 1 compares patients who had dd-cfDNA ≤ 1 initially and throughout their period of follow-up and consisted of 62/106 (58.5%) patients to those with dd-cfDNA > 1 who constituted 44/106 (41.5%) patients. When compared to patients with dd-cfDNA ≤ 1, 59.0% (26/44) of patients with dd-cfDNA > 1 had BPAR and BKV nephropathy (OR 13.5, 95%CI 4.6,38; *p* < 0.0001), and 88.1% (37/44) had de novo anti-HLA antibodies (OR 60.3, 95%CI 17.2,192.2; *p* < 0.0001). Median dd-cfDNA fraction in those with dd-cfDNA ≤ 1 was 0.29 (IQR 0.20,0.37) and was significantly different from those with dd-cfDNA > 1 (*p* < 0.0001) which was 2.75 (IQR 1.7,4.3).

### Results of kidney biopsy and dd-cfDNA values

**Fig. 3:**
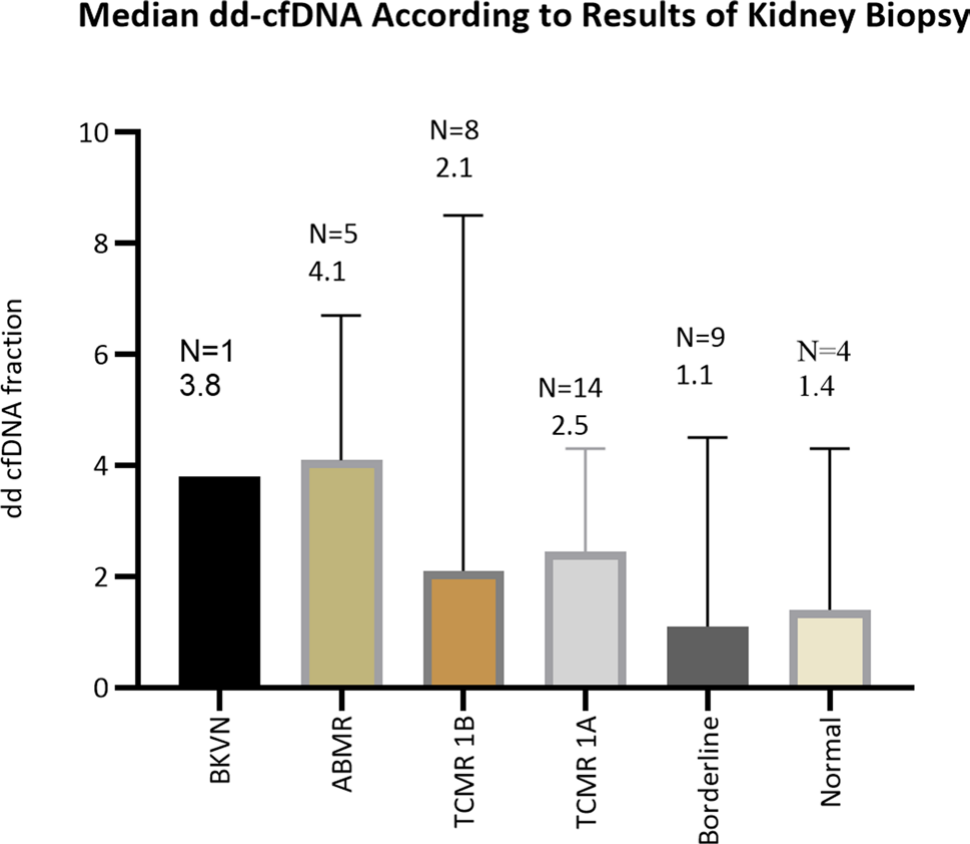
dd-cfDNA for each biopsy classification by BANFF 2019 criteria. Patients with TCMR 1B and ABMR and one patient with BK virus nephropathy had higher dd-cfDNA compared to other categories. Some patients with normal and borderline biopsies had high dd-cfDNA because of the presence of de novo anti-HLA alloantibodies

There were 41 kidney biopsies performed in 36/106 (34.0%) patients, of which 32 had BPAR and 4 had normal biopsies. Of the 32 patients with BPAR, 5 had 2 biopsies. Results are displayed in relation to the median value of dd-cfDNA fraction in each category as seen in Fig. 3. Dd-cfDNA was performed at a median of 38 days (IQR:11,62) before the kidney biopsy. Indications for performing a kidney biopsy included persistently elevated serum creatinine > 20% from baseline or progressively increasing proteinuria. Although not statistically significant because of considerable variation, dd-cfDNA was 4.1 (IQR:2.5,6.6) in ABMR, 2.1 (IQR:0.99,3.5) in TCMR 1B, and 2.5 (IQR:1.15,3.7) in TCMR 1 A and was higher compared to patients with borderline rejection and normal biopsies in which the median dd-cfDNA was 1.1 (IQR:0.64,3.6) and 1.4 (IQR:0.73,3.6), respectively, as seen in Fig. 3. There was one patient with BKV nephropathy with a dd-cfDNA of 3.8% and was highly sensitized before transplant. Six of nine biopsies with borderline rejection and three of four with normal kidney biopsies had de novo anti-HLA antibodies and dd-cfDNA > 1.0. BPAR was more likely to occur in association with de novo HLA antibodies (OR 11.1: 95%CI: 4.0,27.2).

*Dd-cfDNA in highly sensitized patients*: Five patients had pre-formed anti-HLA alloantibodies from being highly sensitized. Three of five highly sensitized patients had DSAs, of which one had BKV nephropathy and the other 1 A TCMR. The remaining patients (as seen in Fig. 2) who were highly sensitized had lower dd-cfDNA than those who had de novo ant-HLA antibodies (0.59 versus 2.2; *p* = 0.001). All patients with ABMR also had TCMR.

### De novo anti-HLA antibodies, DSAs, and dd-cfDNA results

Twenty-six patients had de novo anti-HLA alloantibodies during initial testing*.* On longitudinal follow-up, 16 more developed de novo anti-HLA alloantibodies resulting in a total of 42 patients. DSAs were observed in 33/42 (78.6%) patients with de novo anti-HLA antibodies. Median dd-cfDNA of all patients who developed de novo anti-HLA antibodies were 2.6 (IQR 1.3,4.0). Patients with DQ and DR de novo anti-HLA antibodies (OR 115.2: 95%CI 24.8, 509.5; *p* < 0.0001) and those with de novo DSAs (OR 50.8: 95%CI 13.0, 168.7; *p* < 0.0001) were highly likely to have dd-cfDNA > 1, as described in Table 1. There were 27 kidney biopsies (Fig. 2) done in 24/42 (66.7%) children who had de novo anti-HLA antibodies. Five had ABMR, 5 had TCMR 1B, and 7 had TCMR 1 A. Seven had borderline rejection (median dd cfDNA 1.1), and three had normal biopsies (median dd cfDNA − 1.4). Eighteen patients with de novo anti-HLA alloantibodies did not have biopsies as their eGFR did not change during the observation period. They had their baseline immune suppression increased.

De novo anti-HLA alloantibodies were associated with pyelonephritis in 3 and viral illnesses in 3. Twenty-nine of forty-two patients (69%) with de novo anti-HLA alloantibodies had HLA DQ, 1/42 (2.4%) had DP, 6/42 (14.3%) had DR, and 6 (14.3%) had class 1 antibodies.

### Sequential analysis of dd-cfDNA and eGFR

During the follow-up period, 97 patients had a second and 79 had a third dd-cfDNA testing done. Patients who had never had BPAR or de novo anti-HLA antibodies throughout the follow-up period were compared with patients with BPAR and with those with de novo anti-HLA antibodies but no BPAR. A repeated measures linear mixed effect model (Table 2) with the reference group being those with de novo antibodies without BPAR revealed a significant difference in dd-cfDNA between those with no antibodies or BPAR (*p* < 0.0001). Dd-cfDNA differed significantly in those with HLA antibodies (whether they were DSA positive or negative) from those who had no rejection or HLA antibodies. Although dd-cfDNA values gradually declined in those with de novo anti-HLA antibodies, patients continued to have dd-cfDNA > 1 which was associated with high level DQ antibodies on follow-up.

**Table 2:** Linear mixed model for repeated measures

Groups	dd-cfDNA^1^	
	Effect estimate^2^	*p*-value
No BPAR has HLA antibodies (reference)		
No BPAR, no HLA antibodies	0.085	< 0.001
BPAR^3^	1.548	0.161
HLA antibodies, DSA, and rejection		
No BPAR, no HLA antibodies (reference)		
Have HLA antibodies, DSA negative	10.455	< 0.001
Have HLA antibodies, DSA positive	13.035	< 0.001
BPAR^3^	18.289	< 0.001

^1^Mixed model for repeated measures

^2^The ratio of mean dd-cfDNA in the group of interest over the reference group

^3^For BPAR, dd-cfDNA closest to the date of biopsy was used; for no BPAR groups, all dd-cfDNA were included

The mean dd-cfDNA score was 91.5% lower in the group with no BPAR or HLA antibodies compared to the group with no BPAR but had presence of HLA antibodies

**Fig. 4:**
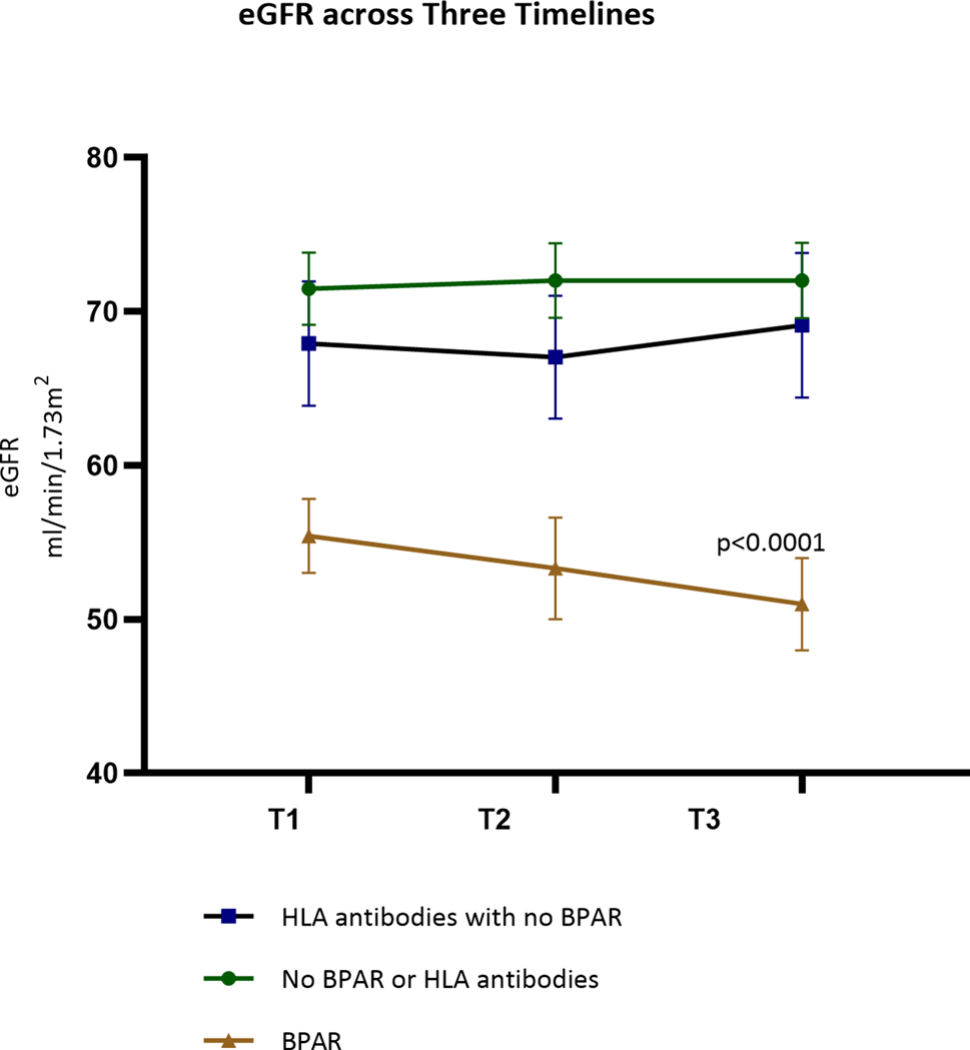
Comparison of eGFR in patients with no BPAR or de novo HLA antibodies with those with de novo HLA antibodies and no BPAR, and those with BPAR. eGFR was trended at three timepoints corresponding to dd-cfDNA testing. The initial dd-cfDNA was done at 3.5 ± 3.7 years post-transplant. The follow-up period for dd-cfDNA testing was 10.3 ± 7.3 months between time 1 (T1) and time 2 (T2) and 7.7 ± 4.7 months between T2 and time 3 (T3). There was a significant difference in eGFR between those who had BPAR versus other categories

Longitudinal assessment of eGFR with a follow-up period of 10.3 ± 7.3 months between the first and second dd-cfDNA test and 7.7 ± 4.7 months between the second and third dd-cfDNAs in those who never had rejection or de novo HLA antibodies versus those with de novo HLA antibodies without BPAR versus those with BPAR is displayed in Fig. 4. At the third time point, mean (SE) eGFR was 72 ± 2.4 mL/min/1.73 m^2^ in those with no BPAR or HLA antibodies versus 66.8 ± 4.7 mL/min/1.73 m^2^ in those with de novo HLA antibodies without clinical evidence of rejection versus 51 ± 3 mL/min/1.73 m^2^ in patients with BPAR and was significantly different between those with BPAR versus the other categories, as seen in Fig. 4.

## Discussion

Donor-derived cell-free DNA (dd-cfDNA) has emerged as a non-invasive biomarker in kidney transplant monitoring [13, 20] that provides insights into the health of the transplanted kidney by detecting fragments of DNA released from the allograft into the recipient’s bloodstream [16, 20]. An elevated fraction of dd-cfDNA is indicative of graft cell injury. In immune quiescent states, the fraction of cell-free DNA derived from the donor is minimal, thereby making it a potentially useful biomarker to distinguish between immune activation and quiescence. It is most useful in the prediction of higher grades of TCMR (greater than 1 A) and ABMR as validated by many studies [10–13, 21, 22]. At our center, we studied its performance characteristics when done for a clinical indication in a cohort of ethnically diverse children who were transplanted and followed for an average of 3 years. In patients where rejection was unlikely, the median dd-cfDNA fraction was 0.25 and remained low throughout the follow-up period unless there was rejection or development of anti-HLA antibodies.

While the association of DSAs with higher dd-cfDNA has been reported by others [11, 12], an important finding in our study was that the appearance of de novo anti-HLA alloantibody with or without DSAs resulted in higher dd-cfDNAs. Three of four patients in our study with normal biopsies had de novo class 2 HLA antibodies with dd-cfDNA > 1. A substantial proportion of the de novo anti-HLA alloantibodies were HLA DQ and DR which were most often associated with DSAs. It has been known that the presence of de novo DQ antibodies is associated with poor response to treatment and decreased graft survival [23, 24]. One can speculate that the appearance of de novo anti-HLA antibodies and a rise in dd-cfDNA may represent an early sign of immune activation even in those with biopsies that revealed no rejection.

The incidence of acute rejection is highest in the first 6 months after transplant but has slowly decreased over the last few years with the use of newer immunosuppressive drugs [25]. Many centers perform protocol kidney biopsies to screen for sub-clinical rejection in the first post-transplant year. The utility of performing protocol kidney biopsies in the first year of transplantation in the current era has been debated [25, 26]. Protocol kidney biopsies have not been shown to improve long-term graft survival although they can be useful in treating sub-clinical acute rejection [26, 27]. In one study, Kanzelmeyer et al. concluded that protocol biopsies were associated with change in treatment only if associated with a > 20% decrease in kidney function [27]. There are also similarities in histological phenotypes with multiple types of injuries provoking T cell-mediated inflammation making the distinction between rejection and infection difficult, necessitating other molecular diagnostic tools [28]. Moreover, in recent years, it has been recognized that the incidence of late acute rejection has increased, partly from the occurrence of de novo anti-HLA antibodies and nonadherence, necessitating serial monitoring and use of non-invasive monitoring tools [23, 29, 30].

Therapeutic drug monitoring is the mainstay of immunosuppressive drug therapy in transplant recipients. Based on clinical trials and observations of improving acute rejection rates after transplant, optimal immunosuppressive drug levels have been recommended [8]. Although monitoring of drug levels has been the standard of management to prevent rejection, individual determination of the intensity of immunosuppression has not been well-studied. Infections and other complications related to immunosuppressive drug therapy contribute to AKI, lymphoproliferative disease, and graft loss, which often leads to morbidity and mortality in children [31–35]. Increased rehospitalization rates in pediatric kidney transplant recipients are largely attributed to infections [35]. The risk of post-transplant viral and bacterial infections is related to the intensity of immunosuppression [36, 37]. Severe infections can also cause renal parenchymal injury and increase dd-cfDNA [38]. Therefore, clinical context is important for the interpretation of dd-cfDNA, making it an important limitation. Dd-cfDNA cannot be used as a stand-alone test and, when elevated, has to be interpreted with other clinical information and combination of tests, such as presence of infections, tacrolimus intra-patient variability (TacIPV), magnitude of viral replication and virus-specific T cell monitoring (to assess host immunity to the virus), and the appearance of de novo antibodies, for clinical decision-making [39, 40]. Studies suggest that monitoring the viral load of torque teno virus may help with risk stratification of rejection versus infection after solid organ transplantation [37].

In clinical situations such as EBV and BKV infection, immunosuppression reduction is the first line of management. However, it is not easy to determine the duration of reduction in immunosuppressive drug therapy, and immune reactivation is dependent on the host’s immune status. Dd-cfDNA may potentially help establish immune quiescence in patients on lower-than-recommended immunosuppression and preemptively detect signs of immune activation. Other promising biomarkers for non-invasive monitoring are urinary microRNA and AlloMap with specific gene expression signatures for sub-clinical rejection and immune quiescence respectively, and serum cytokines [41–43]. Mitochondrial cell-free DNA and highly sensitive C reactive protein are reported to be correlates for vascular fibrosis [44].

An important observation in our study which has not been previously reported is that the development of de novo anti-HLA antibodies was highly associated with an increase in dd-cfDNA. In our case series, there was a higher likelihood of dd-cfDNA being > 1 when rejection was associated with anti-HLA antibodies and DSAs. In contrast, other studies validate the usefulness of dd-cfDNA in predicting rejection with TCMR > 1 A [10–12, 15, 21, 22].

The limitation of our study is that only 35% of patients had kidney biopsies done. Therefore, in those without kidney biopsies and low dd-cfDNA, it was assumed that there was no underlying parenchymal injury in the transplanted kidney, which was strengthened by the fact that it was associated with stable eGFR through three time periods. Moreover, it would have been impractical to perform kidney biopsies on all patients. Currently, the practice in many centers, including ours, is to perform kidney biopsies for a clinical indication such as AKI or high-grade proteinuria after the first post-transplant year. Until the adoption of universal biopsies is agreed upon, or tested in a clinical trial in a cohort of pediatric patients, our data suggests that dd-cfDNA can be used as an adjuvant monitoring tool in addition to surveillance with DSA and other clinical tools, such as intrapatient variability in tacrolimus (TacIPV), to optimize immunosuppression [26, 27]. Our data suggests that the best utility of dd-cfDNA in pediatric kidney transplantation is in surveillance, building upon previous clinical validation for rejection detection. However, there are limitations to its use as a continuous monitoring tool for the pediatric population, and rejection may still occur due to various factors, including medication nonadherence, after a dd-cfDNA test with low levels. Moreover, the cost of the test and the relatively large blood volume required (5 mL) in pediatric patients further necessitate a structured surveillance approach tailored to different clinical scenarios and based on changes in risk. Our proposed surveillance strategy for this population is to perform dd-cfDNA for surveillance every 3 months in the first year and every 3–6 months in the second and third post-transplant years. If no significant changes are made in immunosuppression, and if a patient is known to be adherent to medications, consider testing every 6 months thereafter. Additionally, specific clinical indications, as outlined in this manuscript and the corresponding consort diagram, should prompt additional dd-cfDNA testing.

In conclusion, dd-cfDNA is an adjunct tool that can be used as a serial biomarker for kidney injury and rejection. Elevated dd-cfDNA is highly associated with rejection and presence of class II antibodies and DSAs. Low median values of 0.25 are observed in immunoquiescent states. Our study indicates that the best utility of this molecular biomarker is in surveillance and can be leveraged for non-invasive clinical decision-making. Beyond rejection risk assessment, our study highlights the role of dd-cfDNA in guiding immunosuppression modulation. A multimodal approach that integrates dd-cfDNA with serum and urinary gene transcripts for rejection and BKV capsid protein mRNA may enhance diagnostic accuracy and improve clinical decision-making, thus narrowing the gap towards achieving precision medicine and determining optimal doses of immunosuppression in pediatric kidney transplant recipients [41, 42, 43, 45, 46].

## Supplementary Information

Below is the link to the electronic supplementary material.

Graphical abstract (PPTX 120 KB)

## Data Availability

The data that support the findings of this study are available on request from the corresponding author. The data is not publicly available due to privacy restrictions.
