# Supplementary material for: Donor-derived cell-free DNA testing in pediatric kidney transplant recipients: indications and clinical utility
Source: Pediatr Nephrol. 2025 Apr 14;40(8):2669–78. doi: 10.1007/s00467-025-06770-w (PMC12187795; doi:10.1007/s00467-025-06770-w)
Supplement: Supplementary file 1 — Graphical abstract (PPTX 120 KB) [file 467_2025_6770_MOESM1_ESM.pptx]

## Slide 1
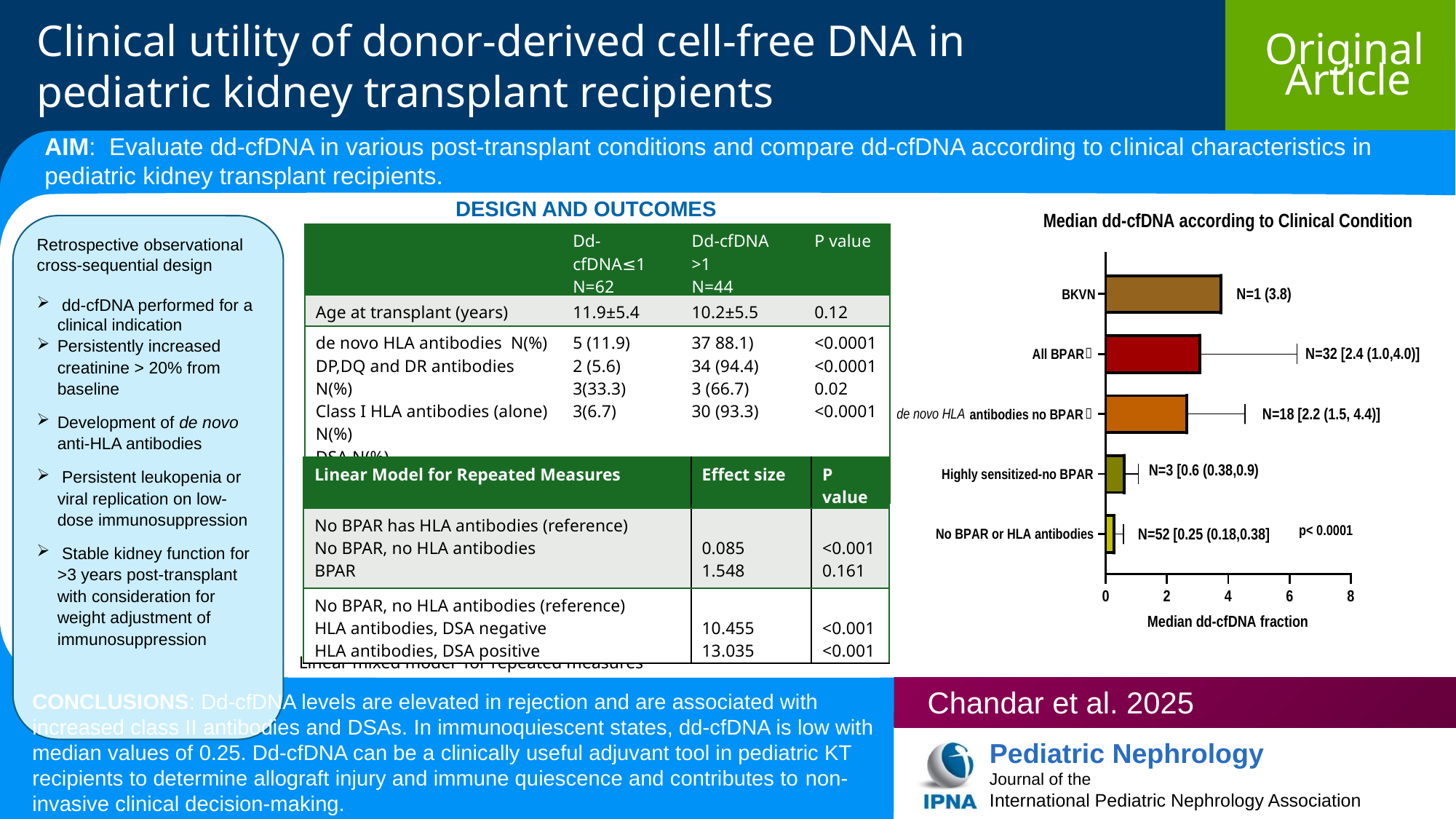

Clinical utility of donor-derived cell-free DNA in
pediatric kidney transplant recipients
AIM: Evaluate dd-cfDNA in various post-transplant conditions and compare dd-cfDNA according to clinical characteristics in pediatric kidney transplant recipients.
DESIGN AND OUTCOMES
Retrospective observational cross-sequential design
 dd-cfDNA performed for a clinical indication
Persistently increased creatinine > 20% from baseline
Development of de novo anti-HLA antibodies
 Persistent leukopenia or viral replication on low-dose immunosuppression
 Stable kidney function for >3 years post-transplant with consideration for weight adjustment of immunosuppression
| | Dd-cfDNA≤1 N=62 | Dd-cfDNA >1 N=44 | P value |
| --- | --- | --- | --- |
| Age at transplant (years) | 11.9±5.4 | 10.2±5.5 | 0.12 |
| de novo HLA antibodies N(%) DP,DQ and DR antibodies N(%) Class I HLA antibodies (alone) N(%) DSA N(%) | 5 (11.9) 2 (5.6) 3(33.3) 3(6.7) | 37 88.1) 34 (94.4) 3 (66.7) 30 (93.3) | <0.0001 <0.0001 0.02 <0.0001 |
| BPAR and BKVN N (%) | 6 (18.8) | 26 (81,2) | 0.0001 |
| Linear Model for Repeated Measures | Effect size | P value |
| --- | --- | --- |
| No BPAR has HLA antibodies (reference) No BPAR, no HLA antibodies BPAR | 0.085 1.548 | <0.001 0.161 |
| No BPAR, no HLA antibodies (reference) HLA antibodies, DSA negative HLA antibodies, DSA positive | 10.455 13.035 | <0.001 <0.001 |
Linear mixed model for repeated measures
Chandar et al. 2025
CONCLUSIONS: Dd-cfDNA levels are elevated in rejection and are associated with increased class II antibodies and DSAs. In immunoquiescent states, dd-cfDNA is low with median values of 0.25. Dd-cfDNA can be a clinically useful adjuvant tool in pediatric KT recipients to determine allograft injury and immune quiescence and contributes to non-invasive clinical decision-making.
